# Distractor-induced saccade trajectory curvature reveals visual contralateral bias with respect to the dominant eye

**DOI:** 10.1038/s41598-022-26319-3

**Published:** 2022-12-16

**Authors:** Romain Chaumillon, Nadia Alahyane, Patrice Senot, Christelle Lemoine-Lardennois, Karine Doré-Mazars, Dorine Vergilino-Perez, Alain Guillaume

**Affiliations:** 1 Laboratoire Vision Action Cognition, Université Paris Cité, EA 7326, Boulogne Billancourt, France; 2 Centre National de La Recherche Scientifique, CNRS, Paris, France

**Keywords:** Saccades, Human behaviour

## Abstract

The functional consequences of the visual system lateralization referred to as “eye dominance” remain poorly understood. We previously reported shorter hand reaction times for targets appearing in the contralateral visual hemifield with respect to the dominant eye (DE). Here, we further explore this contralateral bias by studying the influence of laterally placed visual distractors on vertical saccade trajectories, a sensitive method to assess visual processing. In binocular conditions, saccade trajectory curvature was larger toward a distractor placed in the contralateral hemifield with respect to the DE (e.g., in the left visual hemifield for a participant with a right dominant eye) than toward one presented in the ipsilateral hemifield (in the right visual hemifield in our example). When two distractors were present at the same time, the vertical saccade showed curvature toward the contralateral side. In monocular conditions, when one distractor was presented, a similar larger influence of the contralateral distractor was observed only when the viewing eye was the DE. When the non dominant eye (NDE) was viewing, curvature was symmetric for both distractor sides. Interestingly, this curvature was as large as the one obtained for the contralateral distractor when the DE was viewing, suggesting that eye dominance consequences rely on inhibition mechanisms present when the DE is viewing. Overall, these results demonstrate that DE influences visual integration occurring around saccade production and support a DE-based contralateral visual bias.

## Introduction

Eye dominance, the preference for one eye in monocular tasks^1^, forms a lateralization of the visual system. Supposedly first described in the sixteenth century^2^ (see^3,4^), its systematic study began in the early twentieth century^3,5^. The Meta-analysis of Bourassa et al.^6^ established that 34.5% of right-handers have a left dominant eye (DE). Minucci and Connors^7^ demonstrated that in a monocular simple button-press task, reaction times were shorter when using the DE compared to non dominant eye (NDE). This was confirmed by Coren and Porac^8^ and Coren^9^. Later, Shneor and Hochstein^10,11^ demonstrated that the DE has an advantage in more elaborate tasks like visual feature or conjunction search, which has also been verified recently by Liu et al.^12^. At the neuronal level, stimulation of the DE resulted in higher and shorter visually evoked potentials when using electroencephalography^13^ (see also^14^) and in larger visual areas activation when using functional imagery^15^. Importantly, the magnetoencephalography study by Shima et al.^16^ showed that, in monocular condition, only the stimulation of the temporal retina induced larger visual activations when the DE and the NDE were compared. Evoked potentials when the nasal retina was stimulated were not different between the two eyes. Authors concluded that eye dominance was controlled by the ipsilateral hemisphere.

Interestingly, the suggestion that the eye dominance is controlled by the ipsilateral hemisphere is consistent with our previous behavioral results showing that, in binocular condition, hand reaction times are significantly faster for stimuli presented in the contralateral visual hemifield with respect to the DE, and hence processed by the temporal retina of the DE^17,18^. Tagu et al.^19^ studied the influence of the DE in a simple saccadic task using distractors: if a distractor appears close to and at the same time as a saccadic target, the saccade often lands in between the distractor and target positions, a phenomenon referred to as the global effect (see e.g.^20^). They observed a reduced global effect when visual information was presented in the contralateral hemifield with respect to the DE, which they attributed to better processing abilities for this hemifield (but see^21^). Finally, a left eye dominance has been shown to reduce the pseudoneglect phenomenon^22^ and the leftward bias classically observed for faces processing^23^. All these results support a contralateral bias associated with the eye dominance (Older studies cited above and reporting faster RT for the DE^7–9^ do not bring information regarding this bias associated with the eye dominance as they were conducted in foveal vision).

The aim of the present work was to further assess the hypothesis of a visual contralateral bias with respect to the DE. We reasoned that a sensitive method to evaluate this eye dominance influence would be to study saccade trajectory curvature induced by the unexpected appearance of a visual distractor in a simple saccade to target task. Indeed, this method is acknowledged as a valuable measure of a distractor strength in different saccadic tasks (see for review:^24,25^). Typically, when a visual distractor is presented approximately at the same time as a target for a saccadic eye movement, the saccade toward this target shows a curvature of its trajectory toward or away from (see below) the distractor^26,27^ (see^24,25^ for reviews). We hypothesized that if the DE is associated with more efficient processing for some parts of the visual field, then saccade curvature should be larger when a distractor appears in these specific parts of the visual field.

Saccade trajectory deviation induced by a distractor is related to saccade latency: when saccades are initiated fast, they tend to deviate toward the distractor whereas when they are initiated with longer latency, they tend to deviate away^27–30^. The transition between deviation toward or away from the distractor occurred at a saccade latency of around 200 ms (see e.g.^27^). Initial direction of a saccade is proposed to result from the integration of the distributed activity present on visuomotor maps near the time of saccade onset (population coding^25,31^). A deviation toward the distractor has been seen as a non-resolved target selection^25,32^. When two simultaneous visual stimuli (for example a target and a distractor) induce two activated loci on visuo-motor maps, a completed target selection process results in only one remaining activation (corresponding to the target). For short latency saccades, this selective process could be incomplete due to a lack of time: the contribution of the unsuppressed distractor activation on visuo-motor maps influences the initial direction of the saccade which will show a trajectory curved toward this distractor location^25,32–34^. Alternatively, if the saccade is generated later, the system has the time to inhibit the distractor-related activity. Such a localized inhibition on a visuomotor map would result in a deviation away from the distractor through the read-out of the population activity distribution^25,30,35^ (but see^36^).

**Figure 1:**
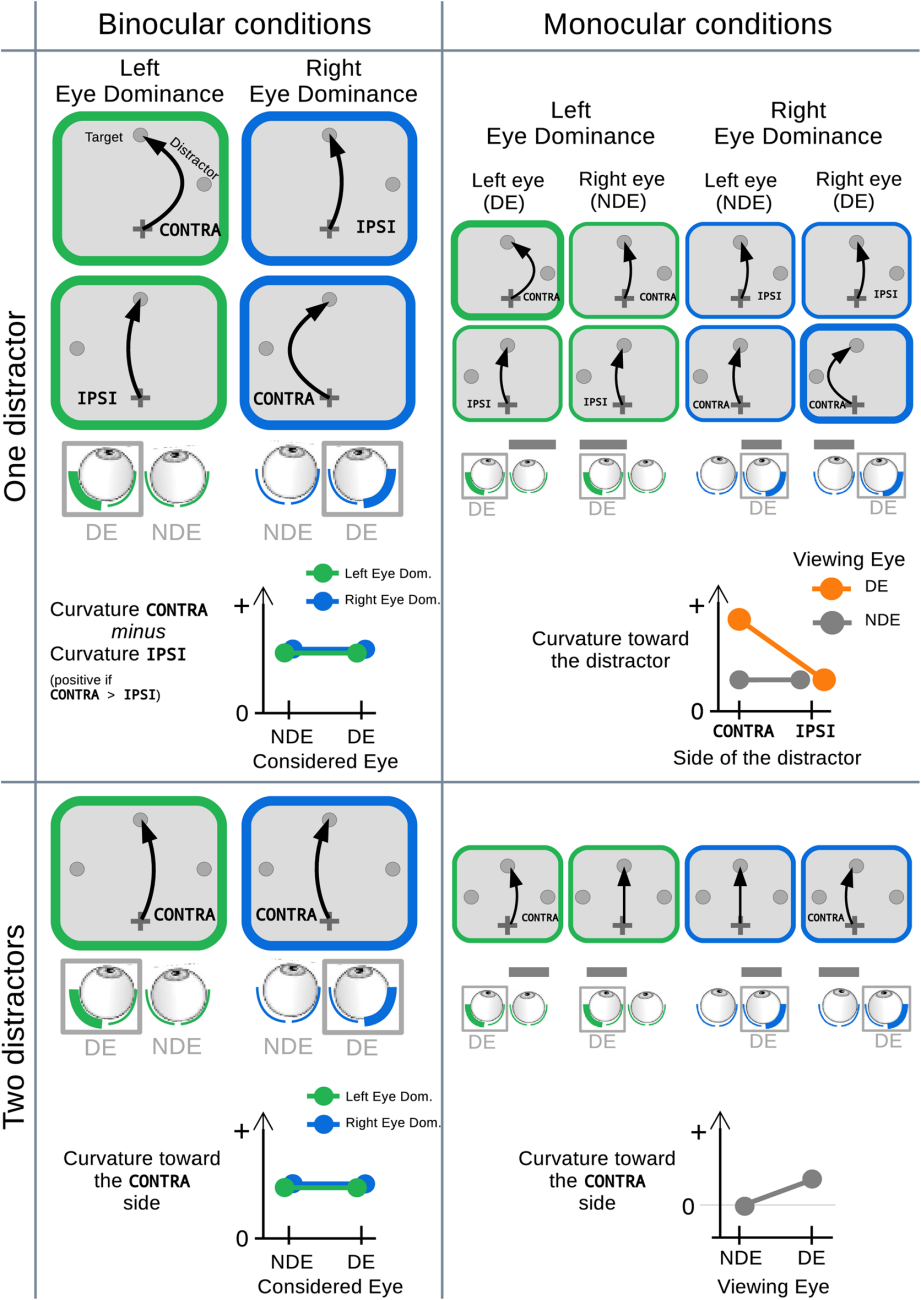
Schematic diagrams showing, for each tested condition, spatial locations of both the saccade Target and the Distractor(s), and predicted saccade curvature according to the hypothesis of a visual bias for the contralateral hemifield with respect to the dominant eye (DE). The predictions concern only the short latency saccades and thus effect of eye dominance on saccade curvature toward the distractor. Larger saccade curvatures should be observed toward a distractor in the contralateral hemifield (CONTRA) with respect to the DE, than toward a distractor in the ipsilateral hemifield (IPSI). Green corresponds to Left eye dominance and blue to Right eye dominance. On the schemes of the two eyes, grey box indicates the DE and the postulated specific status of the DE temporal retina is symbolized by a thick line hemi-retina. NDE corresponds to non dominant eye. In binocular conditions (left side of the Figure), left and right eye dominance are considered separately. In monocular conditions (right side of the Figure), left and right eye dominance results are combined and curvatures are expressed as values for the viewing eye (DE or NDE). The horizontal grey bar represents the opaque patch in front of one eye. The hypothesis of a visual bias toward the contralateral hemifield due to a specific temporal retina organization in the DE leads to the following predictions. In the Binocular/One distractor condition (upper-left), curvature CONTRA minus curvature IPSI should always be positive. In the Binocular/Two distractors condition (lower-left), a slight curvature toward the contralateral distractor should be observed. In the Monocular/One distractor condition (upper-right), curvature toward the distractor should be larger than other conditions only when the viewing eye is the DE (orange) and the distractor is in the contralateral hemifield. In the Monocular/Two distractors condition (lower-right), curvature should be slightly toward the distractor in the contralateral hemifield only when the viewing eye is the DE. See text for further details.

Little is known about the mechanism underlying the effect of eye dominance (e.g.^37^). Besides the results showing that eye dominance is associated with larger and faster visual activations^13–16^, eye dominance could also influence more elaborated and integrated processes such as inhibitory processes at play during saccadic target selection. To remain with a straightforward scheme and rely solely on results showing larger visual activations, we designed the present study to evaluate the eye dominance influence on the curvature toward a distractor in short latency saccades: if eye dominance is associated with larger visual activations for the distractor then larger saccade curvature toward this distractor should be observed. We focused on the situation of toward-the-distractor deviation by using a gap paradigm that favors short latency saccades^38^. Moreover, vertical saccades are de facto the best condition to test the ipsilateral *vs.* contralateral hemifield difference in visual distractor influence. This choice was also supported by the fact that vertical saccades are prone to greater distractor interference than horizontal saccades^39^. In this context of short latency saccades, the hypothesis of eye dominance associated with larger activation for stimuli appearing in the contralateral hemifield, through a specific organization of the temporal retina of the DE, leads to clear predictions on saccade trajectory curvature for one-distractor or two-distractors cases, for a classical binocular condition. Importantly, monocular conditions allow to further test the specificity of this temporal retina organization in the DE, compared to the NDE. Predictions corresponding to the tested configurations in the present study are presented on Fig. 1. Nevertheless, before detailing these predictions, the usefulness of the naso-temporal asymmetries framework, in which questions of visual activation are often discussed, should be addressed here.

The monocular condition allows to clearly separate nasal and temporal hemifields. With this approach an advantage for the temporal visual hemi-field (processed by the nasal hemi-retina) was observed in different visuo-motor tasks^40–42^ (see^43^ for a review). This was associated with a potential specificity of the nasal retina^44,45^. Nevertheless, this naso-temporal asymmetry was not always observed^46,47^ (see^48^ for a discussion of this point). Concerning our present study, one should first note that this asymmetry was never evaluated for saccade curvatures induced by the appearance of distractors. More importantly, caution should be taken when considering our hypothesis of a contralateral advantage with respect to the DE within this scheme of naso-temporal asymmetries. Indeed, if we consider for example a participant with a right DE and if we consider his/her right eye, the contralateral hemifield with respect to his/her DE will be the nasal visual hemifield. But when we consider the other (left) eye, the visual hemifield contralateral to the DE will be the temporal one. Thus the framework of naso-temporal asymmetries cannot be adopted to present our predictions and results.

We now detail the predictions entailed by the hypothesis of an advantage for the contralateral hemifield with respect to the DE. Under binocular conditions, with a single lateralized distractor (Fig. 1, top-left), a larger activation for stimuli appearing in the contralateral hemifield should result in a stronger curvature toward the contralateral location relative to the DE. In other words, the difference between CONTRA and IPSI curvature values should always be positive. As conjugate eye movements are controlled by a single visuomotor system, no difference should be observed in the behavior of the two eyes (NDE and DE). With two distractors appearing simultaneously on both sides (Fig. 1, bottom-left), a scenario of no-eye dominance influence would result in a balanced influence of these two distractors and, consequently, a straight saccade trajectory. But, alternatively, if there is an eye dominance influence, saccades should be curved toward the contralateral side with respect to the DE because of a larger distractor influence. Again, no difference should be observed between the two eyes.

Under monocular conditions, with a single lateralized distractor (Fig. 1, top-right), if the DE is stimulated, the contralateral preference should result in a larger curvature toward the contralaterally-presented distractor. Alternatively, if the NDE is stimulated, both the temporal and nasal retina would have the same status, and similar curvature values should be obtained for both distractor sides. Also, curvature values obtained in this last condition should be similar to those observed for the IPSI distractor when the DE is stimulated, because activity evoked by the distractor would be at a basic level, which would be lower than the level obtained for contralateral stimulus seen by the DE. With two distractors in monocular conditions (Fig. 1, bottom-right), with the DE stimulated, the saccade should curve toward the contralateral side. Conversely, the stimulation of the NDE with two distractors should result in a straight saccade as no distractor takes over the other.

Predictions could also be made concerning saccade latency. The gap paradigm used here should elicit short latency saccades^38,49,50^. Additionally, it has been shown that the onset of a visual distractor relatively far from a saccadic target induces a latency increase^51–53^, an effect referred to as the “remote distractor effect”^53^. A further progressive latency increase when going from one distractor to two distractors was also described^41,54^. If, as described above, eye dominance is associated with larger activation for distractors placed in the contralateral hemifield with respect to the DE, a distractor in this hemifield should produce a larger saccade latency increase.

The aim of the present work was to evaluate these predictions on saccade curvature and latency in order to test the hypothesis of a contralateral visual bias induced by eye dominance. We recorded saccade trajectory of both eyes (binocular conditions), or of each eye separately in monocular conditions, while one or two lateralized distractors could appear around target onset.

## Methods

### Participants

Thirty-two right-handed participants (mean age = 25.2 ± 5.3 years, 6 males) were included in this experiment after having provided signed written consent. All participants were healthy, reported normal or corrected-to-normal vision, and showed no sign of neurological disorders. The experimental protocol was approved by the local ethics committee (Université Paris Cité, IRB n°. 20130500001072) and the study was performed in accordance with the ethical standards laid down in the Declaration of Helsinki.

The handedness of each participant was tested by the Edinburgh Handedness Inventory^55^ (score = lateralization quotient). According to this test, a lateralization quotient of + 100% represents extreme right hand preference and a lateralization quotient of − 100% indicates extreme left hand preference; the mean value obtained here was 81.7 ± 17.7% (range: 37–100%). We assessed the eye dominance of participants with the hole-in-card test^56^, which is known to be the most reliable test to determine eye dominance^57^ and is not influenced by handedness. The test was repeated three times, and all the participants were consistent across the three measures (i.e., the hole in the card was aligned with the same eye). Participants recruitment was carried out until we had two groups of 16 participants: 16 participants with a left DE and 16 participants with a right DE. Results from one right-DE participant were not usable due to technical problems, leading to 15 participants for the group with right eye dominance.

### Experimental setup

Each participant was tested in three strictly identical sessions for visual stimulations and task, the only difference being that viewing was Binocular or Monocular (Monocular DE and Monocular NDE). Participants were seated in a dimly lit room, 57 cm away from the screen, with their heads kept stable with a chin and forehead rest. Stimuli were presented on an Iiyama HM240DT monitor (Iiyama, Nagano, Japan) with a refresh rate of 170 Hz and a screen resolution of 800 × 600 pixels. Eyes movements were recorded with an EyeLink 1000^®^ (SR Research, Ontario, Canada) having a spatial resolution of 0.01° and an average accuracy of 0.25°. In Binocular conditions, movements of both eyes were recorded with a 500 Hz temporal resolution. In Monocular conditions, movements of the viewing eye were recorded (500 Hz) while vision with the other eye was blocked through an opaque patch.

### Stimuli and procedure

The order of the three sessions (Binocular, Monocular DE, and Monocular NDE) was randomized across participants. Each session began with a 9-points calibration and then comprised 165 trials involving vertical upward or downward saccades. The distractor could appear only when the target was presented above the fixation point (i.e., for upward saccades). There were 132 trials with an upper target: 33 baseline trials without a distractor, 33 with a distractor in the left hemifield, 33 with a distractor in the right hemifield, and 33 with two distractors at the same time (both on the left and on the right). For 33 other trials, the target appeared below the FP to avoid predictability in target position. The order of presentation of all these trials was randomized for each participant.

Stimuli were presented on a gray background (luminance: 25 cd/m^2^). Each trial started with a white central fixation cross (height: 0.4°; luminance: 35 cd/m^2^). The eye position was checked, and if the distance between the eye position and the center of the cross was greater than 0.75°, the trial was canceled and returned later in the session. After a random time period of 300, 500, or 700 ms, the fixation cross disappeared. The screen remained empty for 200 ms (gap period; see^50^ for the relationship between the fast-saccades frequency and the duration of the gap). A target consisting of a small white circle with a diameter of 0.5 deg (45 cd/m^2^ luminance) then appeared for a period of 1000 ms on the vertical axis, either 10 deg above or 10 deg below the initial fixation cross. Participants were required to perform vertical saccades as quickly and accurately as possible. When present, the distractor(s) appeared 16 ms after target onset for 100 ms. Distractors were placed 5 deg on the left or/and on the right from the middle of the vertical line that would connect the fixation cross to the target (see Fig. 1). They were similar to the target: a small white circle with a diameter of 0.5 deg, but with a luminance of 55 cd/m^2^. Mulckhuyse et al.^30^ and Ludwig & Gilchrist^58^ indeed observed that target/distractor similarity increases saccade curvatures.

### Data processing

Eyelink software identified saccade start- and end-points using 30 deg/s velocity and 8000 deg/s^2^ acceleration criteria. Further processing of saccade parameters was carried out using a custom script developed in MatLab (The MathWorks Inc., Natick, MA). For each trial, latency and curvature of the saccade trajectory were computed. Latency was the time elapsed between target onset and saccade initiation. Saccades with latency inferior to 80 ms or superior to 500 ms were excluded from the analyses to avoid trials in which anticipation or attention failure could have occurred. The curvature of the saccade trajectory was obtained by computing the area under the curve formed by actual saccade trajectory relative to the direct distance between starting fixation position and landing position (see for details:^26,59^). As proposed by these authors, to normalize curvature values across saccades of varying amplitudes, the area under the curve was divided by saccade amplitude which was defined as the shortest distance between saccade startpoint and endpoint. Importantly, as saccade trajectories are never completely straight (e.g.^60,61^), for each participant and session (Binocular, Monocular DE, or Monocular NDE) a baseline was defined by having the mean of curvature for the no distractor condition. Final curvature value for each trial was then obtained by calculating the difference between observed curvature and the baseline (see^26,27,39,58^ for a similar procedure). We provide in the Results section these baseline values with the maximum deviation, in deg with respect to the straight line connecting starting and landing positions, to which they correspond.

### Statistical analyses

**Figure 2:**
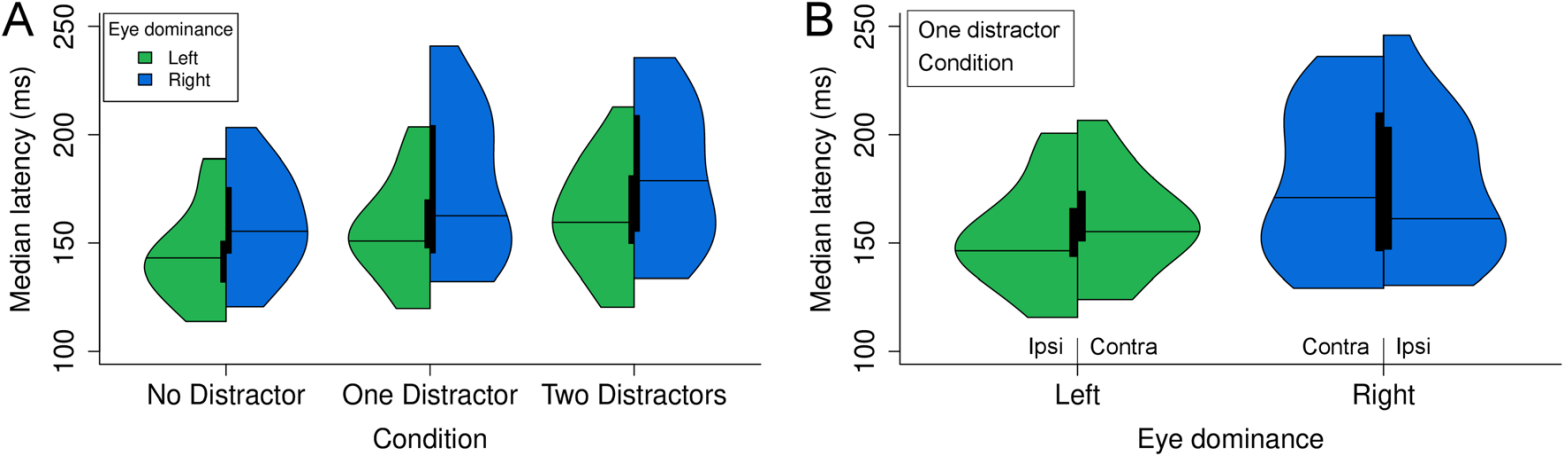
Median latency distributions for both left and right eye dominance participants. (**A**) No Distractor, One Distractor and Two Distractors conditions. Horizontal lines correspond to the median, thick vertical lines show the interquartile range and green and blue area correspond to the kernel latency density estimation (interrupted at minimum and maximum values). For the One Distractor condition, trials with the distractor in the contralateral and the ipsilateral hemifield have been merged. (**B**) One Distractor condition for each participant group. Ipsi and Contra correspond to the hemifield in which the distractor was placed relative to the DE. See text for further details.

All data modeling was conducted using R (R Development Core Team, 2017). A first set of models was used to describe the distractor influence on saccade latency. This description was limited to Binocular sessions. Median latency was computed for each condition (no distractor, distractor on the left, distractor on the right, two distractors) in each participant. Given the non-normality of median distributions (see Fig. 2), we used Aligned Rank Transformations (ART, with the ARTool package in R^62,63^) to conduct mixed effects ANOVAs^64^. This approach allows to compute post-hoc pairwise comparisons with the *art.con* function of the ARTool package. A second set of models focused on saccade curvature and was conducted for Binocular and Monocular sessions. Mean curvature value for each participant and each condition were entered in mixed effects linear regression models^65^ using lmerTest package in R^66^. With two categorical factors with two levels each, main effects and interaction were obtained with setting factor levels to − 0.5 and 0.5. Simple effects of one factor for one level of the other factor were obtained by setting levels of the second factor to 0 and 1. In both cases, mixed effects ANOVA models (latency) and mixed effects linear regressions (curvature), considered factors and designs are detailed in the Results section. Moreover, in both cases, given the “Within-Participants design”, intercepts for Participants were entered as a random effect^67^. Finally, as there is no agreement upon how to calculate standard effect sizes for main effects or interactions in linear mixed models^68^, we simply report differences between conditions to evaluate the effects and provide the 95% confidence interval (CI) for these differences^69^. As a general rule, means were reported ± standard error and with the 95% CI.

We also analyzed the time-course of saccade curvature. We plotted curvature parameters as a function of saccade latency. We then smoothed these curvature-latency curves using a moving Gaussian window between 80 and 300 ms (step size 1 ms and σ = 10 ms) (SMART method^70^; see also^71^). Significant difference between two conditions or with respect to zero were evaluated first with a weighted within-subjects *t* test for each point of the Gaussian smoothed data. Clusters of significant differences were defined as two or more consecutive time points with p < 0.05 and were indicated on the figures by the smoothed curve turning into a thick black line. Then a cluster-based permutation procedure was employed to control for multiple comparisons. Remaining statistically significant clusters after this step were indicated by a star (SMART method^70^).

## Results

### Exclusions

Considering only trials during which one or two distractors could be presented (i.e., with an upward target), we recorded 12,276 trials in 31 participants. Among these trials, 11.92% were discarded from analysis: trials with blinks before or during the saccade (0.02%), eye movements in the wrong direction (6.1%), latency outside the range of 80–500 ms (1.8%), and amplitude differing from 2 standard deviations relative to the mean (4%).

### Saccade latency

Figure 2A shows violin plots of median latency distributions obtained for all the participants in the different conditions of the binocular session. The grand mean of medians across the three conditions was 163.3 ms (± 3.05 ms). A mixed effects ANOVA (Aligned Rank Transform, see "Methods") was employed to study the influence of the DE and Number of Distractors factors. As illustrated in Fig. 2A, there was a small tendency to have shorter latency for left DE than for right DE, but this tendency proved to be statistically non-significant (F (1, 29) = 2.55, p = 0.12). On the contrary, the factor Number of Distractor influenced latency (F (2, 58) = 78.92, p < 0.001): mean median latency was 152.3 ms (± 4.3), 165.9 ms (± 5.5) and 171.6 ms (± 5.5) for the no distractor, one distractor and two distractors conditions, respectively. This effect of the Number of Distractor was similar for the two left and right DE groups (F (2, 58) = 1.12, p = 0.33). Post hoc pairwise comparisons for the different levels of the factor Number of Distractors show that every comparisons were statistically significant from each other (p < 0.0001, Holm method for p values adjustment). Overall latency increased when the saccade system had to resolve a selection conflict induced by the presence of the distractor. This increase in saccade latency was higher when there were two distractors instead of one distractor.

According to the hypothesis tested in the present study, the distractor presented in the contralateral hemifield with respect to the DE should have a stronger influence than the one in the ipsilateral hemifield. A comparison of the contraversive and ipsiversive latency values in the one distractor condition revealed an interaction between the factors Side of the Distractor and DE (Fig. 2B,F (1, 29) = 9.18, p < 0.01): for participants with a left DE, latency was larger when the distractor was in the right hemifield (159.2 vs. 153.7 ms), whereas for participants with a right DE the reverse was observed (174.0 vs. 178.0 ms). Nevertheless post-hoc comparisons showed that this difference was close to the significant level only for the left eye dominance participants (p = 0.07, p = 0.58 for right ED participants).

### Baseline curvature of saccade trajectory without distractor

Values of saccadic curvature reported in the different conditions of the present study are curvature differences with respect to a baseline measured in the no distractor condition (see “Methods”). In binocular condition and for upward 10 deg saccades, in participants with a left eye dominance (n = 450 trials), baseline curvature were − 0.133 deg (± 0.010) [CI − 0.153 − 0.112] and 0.188 deg (± 0.012) [CI 0.164 0.211], for the left and the right eyes respectively. These normalized area under the curve (see “Methods”) correspond to maximum deviations of − 0.23 deg (± 0.017) and 0.29 deg (± 0.021), for the left and the right eyes. In participants with a right dominant eye (n = 447 trials), baseline curvature values were − 0.134 deg (± 0.010) [CI − 0.154 − 0.115] and 0.162 deg (± 0.009) [CI 0.144 0.180], for the left and the right eyes. These normalized area under the curve correspond to maximum deviations of − 0.21 deg (± 0.015) and 0.26 deg (± 0.015).

In monocular conditions, the baseline curvature was − 0.055 (± 0.035) [CI − 0.131 0.021] for the left eye and 0.127 (± 0.024) [CI 0.075 0.179] for the right eye participants with left dominant eye. These values were − 0.032 (± 0.042) [CI − 0.124 0.060] and 0.123 (± 0.033) [CI 0.051 0.195] for the left and right eyes respectively in participants with a right dominant eye.

### Curvature of saccade trajectory as a function of latency

**Figure 3:**
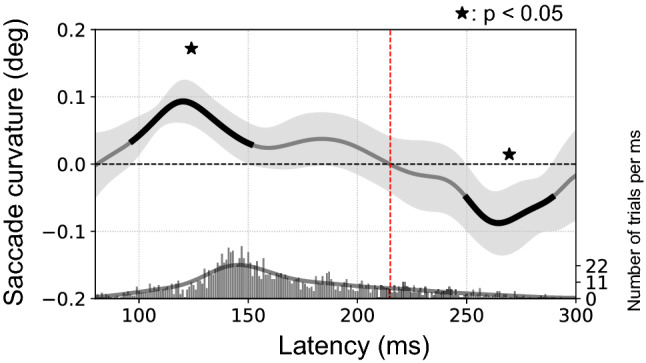
Saccade trajectory curvature as a function of saccade latency. Amount of saccade curvature as a function of latency in the One Distractor / Binocular condition (both ipsilateral and contralateral distractors), averaged across left and right eye dominance groups. Data are smoothed with a Gaussian kernel, with black line segments indicating clusters of time points where a weighted within-subjects t-test resulted in p < 0.05 from zero for the saccade curvature. Asterisks indicate that these clusters survive at the multiple comparisons control at p < 0.05 (see "Methods" for further details). The shaded area is the 95% within-subjects confidence interval. The histogram at the bottom of the graph shows the number of trials per 1 ms bin (right axis, line corresponds to smoothing with the same kernel as above). In agreement with previous studies, curvature was toward the distractor (positive values) for short latency saccades and away from the distractor (negative values) for saccades initiated later. The red dashed line corresponds to the time at which the curvature switch from toward to away (215 ms).

**Figure 4:**
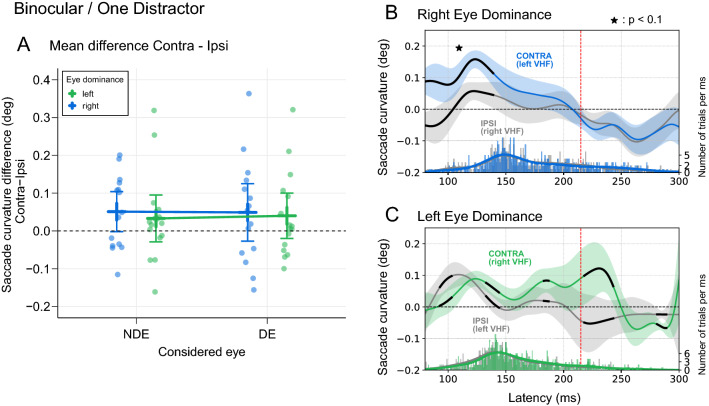
Saccade trajectory curvature in Binocular / One Distractor condition. (**A**) For each participant and for saccades initiated before the toward/away switch latency (215 ms, red vertical line on Fig. 3 and on panels B & C on the right), the difference between curvature values for a distractor in the contralateral hemifield with respect to the DE and for a distractor in the ipsilateral hemifield was plotted as a function of the considered eye. Positive values correspond to a larger curvature for a contralateral distractor. Error bars show 95% confidence intervals. The linear model approach shows a statistically significant difference from zero (p < 0.05) for the whole population. See text for further details. (**B**) Saccade curvature as a function of saccade latency in right DE participants. Blue and grey curves correspond to curvature values for trials with a distractor in the contralateral or ipsilateral hemifield, respectively. Positive values correspond to a curvature toward the distractor. Data are smoothed with a Gaussian kernel, with the black line indicating a cluster of time points where a weighted within-subjects t-test resulted in p < 0.05 between the two hemifields. Asterisk indicates that this cluster survives at the multiple comparisons control at p < 0.1. The shaded areas are 95% within-subjects confidence intervals. The bottom histogram displays the number of trials per 1 ms bin for both conditions (right axis, lines correspond to smoothing with the same kernel as above). (**C**) Saccade curvature as a function of saccade latency in left DE participants. Green and grey curves correspond to curvature values for trials with a distractor in the contralateral or ipsilateral hemifield, respectively. Black line segments correspond to clusters with significant* t* tests between the two hemifields for successive time points, but none of these clusters survive at the multiple comparisons control and thus should be considered as non-significant. Same organization as for panel B.

Curvature of saccade trajectory can be directed toward or away from a distractor depending on saccade latency (see “Introduction”). In the present study, we focused our analysis of the eye dominance influence to the condition in which saccades deviated toward the distractor. Nevertheless, we first checked whether the typically described curvature-latency relationship was found in the present dataset. To allow comparison with previous studies testing only binocular conditions^27,72^, we plotted in Fig. 3 the change of saccade curvature with respect to no distractor values (see “Methods”) when there was one distractor (both in contralateral and ipsilateral hemifield with respect to the DE) across all participants as a function of saccade latency in the binocular condition. A positive value indicates a curvature toward the distractor whereas a negative value corresponds to a curvature away from the distractor. The plot reveals the time course of saccade curvature: a first statistically significant (p < 0.05) cluster of deviation toward the distractor is observed between 100 and 150 ms. The deviation away from the distractor clearly emerges around 240–250 ms. The latency corresponding to the switch between toward and away curvatures is 215 ms (red dashed line), which is very similar to previously obtained values^27,72^. To focus our analysis on the situation of toward-the-distractor deviation, we selected for further analysis only the saccades initiated before the latency corresponding to the switch between toward and away curvature (215 ms). We nevertheless provide for the Binocular/One Distractor condition curvature time course plots containing all the saccades initiated before 300 ms (see Fig. 4B,C). The time course plots for the three other conditions can be found in the Supplementary Material.

### Binocular/One-Distractor condition

As depicted in the upper-left panel of Fig. 1, we predicted that, in the binocular condition, saccade deviation should be larger toward distractors appearing in the contralateral hemifield with respect to DE. As explained above, only saccades initiated before the toward/away latency switch (215 ms) were considered. Nevertheless, an additional important caution was required for this selected set of saccades. As the size of the curvature of saccade trajectory is a function of saccade latency (Fig. 3; see^27^), an accurate comparison of curvature values for distractors presented in the contralateral or ipsilateral hemifield requires similar latency distributions in these two conditions. Indeed, the Fig. 3 shows that a comparison of curvature values between a set of saccades around 125 ms an another set around 175 ms would result in a significant difference. A paired *t* test (across all participants) comparing median latencies of saccades occurring before the limit of 215 ms when the distractor was in the contralateral versus the ipsilateral hemifield revealed no significant difference (t(30) = − 0.34, p = 0.74, CI [− 2.63, 1.88], mean difference = − 0.37 ms).

Our results showed that for both participants groups (with left or right DE), the change of the curvature with respect to control values (see “Methods”) was larger when the distractor was presented in the contralateral hemifield than when it was in the ipsilateral hemifield. As movements of both eyes were recorded, saccade trajectory curvature for each eye was measured and values will be compared. But we first consider here the mean curvature for both eyes. For participants with a left DE (n = 16), the mean curvature toward the distractor was 0.06 (± 0.022) [CI 0.012 0.108] when the distractor was in the contralateral hemifield (right) and 0.023 (± 0.015) [CI − 0.009 0.055] for a distractor in the ipsilateral hemifield (left). For participants with a right DE (n = 15) the mean curvature toward the distractor was 0.07 (± 0.021) [CI 0.025 0.115] when the distractor was in the contralateral hemifield (left) and 0.021 (± 0.025) [CI − 0.033 0.075] for a distractor in the ipsilateral hemifield (right).

For each participant, saccade curvature difference between contralateral and ipsilateral distractors was computed (Fig. 4A). To statistically assess these results, we used a linear mixed model with two fixed effects: Participant Eye Dominance [left eye dominance vs right eye dominance] and Considered Eye [the DE and the NDE]). The Participant number was entered as a random effect (intercept only, see “Methods”). With both fixed factors centered (levels coded − 0.5 and 0.5), the intercept of the model corresponding to the mean difference of deviation (contra-ipsi) was 0.043 (± 0.020) [CI 0.005 0.081] and was clearly different from 0 (t = 2.196, p = 0.036). Neither the factor Participant Eye Dominance, nor the factor Considered Eye modulated this curvature difference (t = 0.039, p = 0.73 and t = 0.195, p = 0.85, respectively). There was no interaction between these two factors (t = 0.288, p = 0.76). In sum, these results show that the curvature of saccade trajectory was always more important when the distractor was in the contralateral hemifield with respect to the DE, regardless of the left or right eye dominance or the considered eye.

Figure 4B illustrates the time course of saccade curvature as a function of latency in participants with right DE. It shows that the difference in saccade curvature between contralateral and ipsilateral hemifields is already present for earlier saccades. This difference then decreases to disappear at around 210 ms. For participants with left DE (Panel C), larger curvature for contralateral distractor appears later but remains above ipsilateral condition even after the 215 ms limit. In both cases (right and left ED), only few saccades were available after the 215 ms limit (mean across all participants = 14, 8% of all saccades occurring before 300 ms, see histograms at the bottom of the graphs). This very small amount of longer latency saccades was not enough to analyze an effect of eye dominance on curvature away from the distractor (see “Introduction”).

### Binocular/Two-Distractors condition

**Figure 5:**
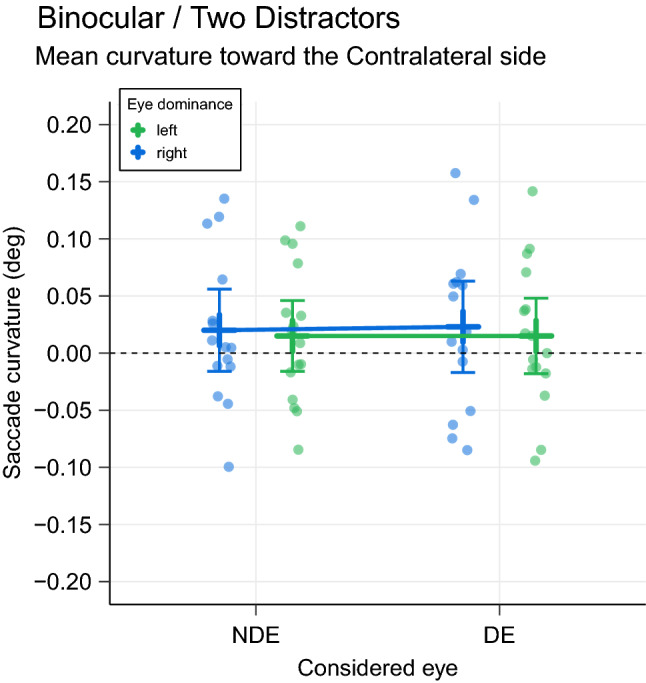
Saccade trajectory curvature in Binocular/Two Distractors condition. For each participant and for saccades initiated before the toward/away switch latency (215 ms, red vertical line on Fig. 3), the saccade curvature values when two distractors were presented was plotted as a function of the considered eye. Positive values correspond to a curvature toward the distractor placed in the contralateral hemifield. Error bars represent 95% confidence intervals. The linear model approach shows a statistically significant difference from zero (p < 0.05) for the whole population. See text for further details.

In the binocular condition with two distractors, according to the hypothesis of no-eye dominance influence, distractors in each hemifield should be processed similarly and the saccade trajectory should be straight. On the contrary, if there is an advantage for the contralateral hemifield with respect to the DE, the influence of the distractor in this hemifield should be slightly larger and a deviation toward this distractor should be observed (Fig. 1, lower-left). The Fig. 5 depicts the results for the two distractor conditions. We found that curvature values were smaller than in single distractor conditions, revealing the competition between the two distractors. Importantly, curvature values always indicated a deviation toward the contralaterally-presented distractor (positive values). In Right eye dominance participants, curvature was 0.023 ± 0.019 [CI − 0.017 0.063] (n = 15) and 0.020 ± 0.017 [CI − 0.016 0.056] (n = 15), for the DE and NDE, respectively. In Left eye dominance participants, curvature was 0.015 ± 0.016 [CI − 0.018 0.048] (n = 16) and 0.015 ± 0.015 [CI − 0.016 0.046] (n = 16), for the DE and NDE, respectively. We fitted a linear mixed model with two fixed effects (Participant Eye Dominance and Considered Eye) and the Participant number as a random effect (intercept only). The grand mean curvature (intercept of the model) was positive and thus toward the contra side (0.018 ± 0.009) [CI 0.001 0.035] and was significantly different from 0 (t = 2.11, p = 0.044). Neither the factor Participant Eye Dominance, nor the factor Considered Eye modulated this curvature (t = 0.38, p = 0.71 and t = 0.08, p = 0.94). There was no interaction between these factors (t = 0.12, p = 0.91). Thus, when two distractors were present at the same time, the curvature of saccade trajectory was directed toward the distractor placed in the contralateral hemifield with respect to the DE, regardless of the type of eye dominance (right or left) or the considered eye (See Supplementary Material—Fig. 1 for the saccade curvature time course plots for this condition).

### Monocular/One-Distractor condition

**Figure 6:**
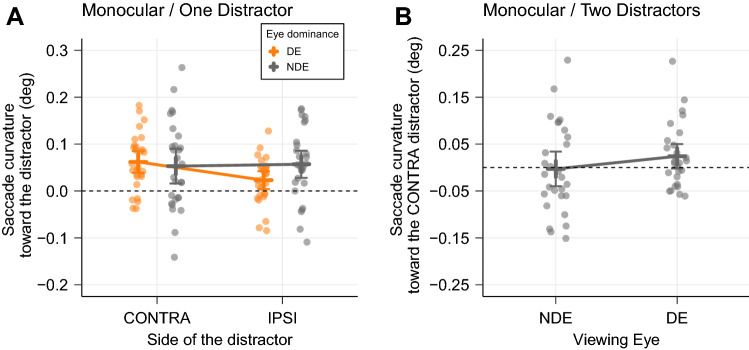
Saccade trajectory curvature in monocular conditions. (**A**) One distractor condition. For both Left and Right eye dominance groups, saccade curvature was measured for contralateral and ipsilateral distractors with respect to the Dominant Eye. (**B**) Two distractors condition. For both Left and Right eye dominance groups, saccade deviation was considered as positive when toward the distractor in the contralateral hemifield with respect to the DE. Error bars represent 95% confidence intervals.

If the eye dominance influence results from a special status of the DE temporal retina (see “Introduction”), then in monocular condition this influence should be observed only when the DE is stimulated. When the NDE is stimulated, curvature should be similar for both hemifields, and values should be similar to those obtained for the ipsilateral hemifield in the DE case (Fig. 1, top-right). Figure 6A shows results when the distractor appeared in the contralateral compared to the ipsilateral hemifield with respect to the DE (left and right eye dominance combined). As before, only saccades initiated before the toward/away latency switch (215 ms, see Fig. 3) were considered. We fitted a linear mixed model with saccade curvature as dependent variable (positive if toward the distractor) and with the Viewing Eye (DE or NDE) and the Side of the Distractor (CONTRA or IPSI) as fixed effects. The Participant number was entered as a random effect (intercept only).

The grand mean of saccade curvature (intercept of the model) was positive and significantly different from 0 (0.048 ± 0.008 CI [0.032 0.065], t = 5.86, p < 0.001), indicating that the curvature was always directed toward the distractor. The first part of our prediction above was verified: when the DE was stimulated (NDE was patched), curvature of saccade trajectory was larger toward the contralaterally-presented distractor (0.062 ± 0.011 CI [0.039 0.085], n = 31) than toward an ipsilaterally-presented distractor (0.023 ± 0.009 CI [0.004 0.042], n = 31). This was a significant difference of 0.040 (t = 2.27, p = 0.027, CI [0.006 0.074]). Our results also confirmed that when the NDE was stimulated (DE patched), curvature were comparable toward the contralateral distractor (0.053 ± 0.018 CI [0.016 0.090], n = 31) and toward the ipsilateral distractor (0.057 ± 0.014 CI [0.028 0.086], n = 31; t = 0.218, p = 0.83). However, contrary to our assumption, these values of curvature when the NDE was stimulated were larger than those observed for the DE/ipsilateral hemifield condition and were instead similar to the DE/contralateral hemifield condition. Indeed, for ipsilateral distractors (IPSI in Fig. 6A), there was a significant difference between the two eyes of 0.034 (t = 1.95, p = 0.05, CI [0.000 0.068]) whereas in the CONTRA condition, the non significant difference between the DE and NDE was only of 0.009 (t = 0.53, p = 0.59, CI [− 0.025 0.043]).

In sum, when the DE was stimulated, curvature was larger when the distractor was presented in the contralateral hemifield. No difference was observed between both hemifields for the NDE, but contrary to our prediction, curvature values were similar to those observed for the DE/contralateral distractor condition (See Supplementary Material—Fig. 2 for the saccade curvature time course plots for these conditions).

### Monocular/Two-Distractors condition

If two distractors are presented at the same time in a monocular condition, we predicted to find a slight curvature toward the contralateral distractor only if the DE is stimulated (Fig. 1, bottom-right). Furthermore, when present, curvature should be smaller than with only one distractor as the two distractors would mutually counteract their influence. Results are illustrated in Fig. 6B. When the DE was stimulated, curvature was slightly toward the contralateral distractor (0.024 ± 0.013 CI [− 0.002 0.05], n = 31). When the NDE was stimulated, curvature was slightly toward the other distractor, in the ipsilateral hemifield (− 0.003 ± 0.018 [CI − 0.04 0.034], n = 31). Nevertheless, given the variability in the measure and the size of the difference between DE and NDE, the probability of obtaining this difference under the null hypothesis was really high (t = 1.198, p = 0.236) (See Supplementary Material—Fig. 3 for the saccade curvature time course plot for this condition).

## Discussion

By studying the influence of lateralized distractors on the trajectory of vertical saccades, we assessed whether sighting eye dominance was associated with a contralateral bias in visual processing. When both eyes were viewing (binocular condition), the curvature of the trajectory of a vertical saccade was larger when the distractor appeared in the contralateral hemifield with respect to the Dominant Eye (DE). If two distractors appeared at the same time in both hemifields, the saccade also slightly, but significantly, deviates toward the contralateral side. When only the DE was viewing (monocular condition), curvature values were also larger for contralateral distractors than for ipsilateral ones. When only the NDE was viewing, curvature values were similar for both distractor positions, but surprisingly, they were as large as those observed for the monocular DE/contralateral distractor condition. Overall, distractors appearing in the contralateral hemifield with respect to the DE had a stronger influence than ipsilaterally-presented ones. These results closely correspond to our predictions based on the hypothesis of a contralateral bias in visual processing with respect to the DE (see Fig. 1).

First, we briefly comment on some observations we made in our control conditions. Several previous studies already reported that saccades were not straight lines toward the target^60,61^. As a baseline, we recorded trajectories in vertical upward saccades for both eyes without any distractor. We show here that saccades from the left eye slightly deviated toward the left hemifield while saccades from the the right eye slightly deviated toward the right. This result represents a pattern that has been noted in individual observations (see Figure 10 of^73^ and Figure 1 of^31^). Further work is required to determine the mechanical and muscular, or nervous origin of this pattern (see for review^74^). Figure 3 provides new information regarding the evolution of saccade curvature induced by the appearance of a distractor as a function of saccade latency (e.g.^27^). The smoothing method employed^70^ allows indeed to more precisely delineate a “toward-the-distractor” zone between 100 and 150 ms, an “away-from-the-distractor” zone from 250 to 300 ms and a transition zone in between. Finally, consistently with previous studies^31,58^, the curvature induced by the distractor was similar for both eyes.

In the different conditions tested in the present study, a larger curvature was found for distractors placed in the contralateral hemifield with respect to the DE. This result is consistent with our previous results depicting significantly shorter reaction times in a simple button-press task when targets appeared in the contralateral hemifield with respect to the DE^17,18^. Such a contralateral bias was also observed for more elaborate emotional stimuli^23^ and in a line-bisection attentional task^22^. Regarding the substrate of this contralateral bias, the MEG study of Shima et al.^16^ highlights the potential role of the temporal retina of the DE as monocular stimulations resulted in larger activations only for the DE.

A contralateral bias and a specific status of the temporal retina could seem counterintuitive at first sight. Indeed, in monocular situations some studies reported a naso-temporal asymmetry consisting in a temporal visual hemifield advantage for behavioral performance such as latency increase induced by a distractor^41^, saccade latencies^42^ or choice saccade to bilateral stimuli^40^. This asymmetry could be associated with a nasal retina superiority. Indeed, the nasal retina sends more axons to the central nervous system than the temporal retina^44,45^. Similarly, in a fMRI study, Toosy et al.^75^ found larger visual activation after nasal retina stimulation in a monocular condition. A primacy of the nasal retina would predict a larger curvature for distractors in the ipsilateral hemifield with respect to the DE, but we found the reverse here. One should note that these naso-temporal asymmetries are still debated (see^43^ for a review, Ref.^48^ for a recent assessment). They are sometimes not observed at all^47^ and have also been hypothesized to be restricted to subcortical structures^46^. Additionally, as underlined in the Introduction, caution should be taken to compare our results to those of studies exploring naso-temporal asymmetries: if results for both eyes are combined, the contralateral visual hemifield with respect to the DE will correspond to both the temporal and the nasal hemifield. This may have obscured results of previous studies^41,42^. Furthermore the specific question of saccadic curvature has never been addressed in this framework of naso-temporal asymmetries. Here we found in monocular condition a larger saccade curvature toward the contralateral hemifield with respect to DE, which corresponds to the nasal visual hemifield. In contrast, we found no difference between the two hemifields for NDE.

The saccade curvature depends on latency: short-latency saccades (≤ 215 ms) deviate toward the distractor, whereas longer-latency ones deviate away from the distractor (see "Introduction", Fig. 3 and^25^ for a review). In the present study, we focused on the deviation toward distractors by selecting only trials in which the saccade was initiated before the latency value of 215 ms. Importantly, after this selection, if trials with a contralateral distractor had shown a constant latency difference with trials with an ipsilateral distractor, this could have explained the observed larger saccade curvature (given the curvature-latency relationship, see Fig. 3). However it was not the case: there was no latency difference between trials with contralateral and ipsilateral distractor positions. We can thus hypothesize on the mechanism that may underlie a larger curvature of saccade trajectory toward the distractor contralateral to the DE. It has been shown that the activation level at the locus corresponding to a distractor on visuomotor maps (superior colliculus or frontal eye field), concurrently with the normal target activation, is related to the size of the saccade curvature toward this distractor^25,32–34,36^. Hence, a possible explanation for the present results is that the distractor would produce a larger activation on visuomotor maps when placed in the contralateral hemifield with respect to the DE. This larger activation would result from its processing by the temporal retina of the DE (see above). Another question concerns the origin of this larger activation. Can it be the result of structural differences between DE and NDE? Some studies indeed found differences between the two eyes^76–78^ but recent works did not replicate these results^79–82^ and argued in favor of a central origin. A general hypothesis about the origin of the contralateral bias is formed below.

Concerning the influence of eye dominance on curvature away from the distractor that occurs for saccades with longer latencies, only preliminary and fragmented observations can be made in our study. As indicated in the Introduction, the present study was specifically designed to examine the eye dominance influence on saccade curvature toward the distractor when saccades are generated with shorter latency by using a gap paradigm. For the few trials that were recorded with latency > 215 ms, it can be seen in participants with right DE that there is no difference in the distractor influence between the two hemifields (Fig. 4B). For the left eye dominant participants (Fig. 4C), one may first note that the difference between the two hemifields for the toward-the-distractor curvature would have been even larger had the switch limit (toward to away) been fixed 40 ms later. For these participants, available results do not allow to discuss the influence of the DE on curvature away from the distractor. Further work is required to study this type of influence.

An interesting point corresponds to results obtained in the monocular condition with a single distractor (Fig. 6A). In the NDE stimulation case, we predicted a smaller curvature than that obtained in the case of the DE stimulation with a contralateral distractor (see Fig. 1, upper-right). Instead, we found that curvature values when the NDE was stimulated, were similar to the values obtained for the contralateral hemifield in the monocular DE condition. A possible interpretation would be that when stimulated, the DE tends to inhibit the vision from the other eye. When the DE is not viewing, this inhibition would be removed and visual activation, notably following a distractor appearance, would be as large as when the DE processes stimulations from the contralateral hemifield. This tendency to inhibit information coming from the NDE is probably at work when one aligns, for example, a hand-held pen with a distant target with both eyes open. Here the inhibition would ensure that information received by the NDE does not hinder the alignment maneuver too much. A similar proposition of DE inhibiting NDE was also made by Shneor and Hochstein in their studies of visual search^10,11^. As underlined by these authors, this means that the eye dominance phenomenon refers not only to the choice of one eye when one has to perform a monocular task, but it also has a role in binocular viewing, at a level of the integration of information coming from both eyes. Importantly, this proposed inhibition of the NDE by the DE does not account for the lateralized effect found here and in previous studies, namely a contralateral bias with respect to the DE. We propose here a speculation on the origin of this bias. From an evolutionary point of view, the alignment mentioned above is notably decisive in tasks like throwing something toward a target (aiming abilities). If we suppose that these alignments were made before the ability to unilaterally close the NDE, then specific mechanisms could have appeared to inhibit the double image occurring in these cases. Note that this double image resides specifically in the ipsilateral visual hemifield with respect to the DE. Could the contralateral bias with respect to the DE be a byproduct of this specific inhibition of the ipsilateral hemifield to attenuate diplopia induced by DE-based alignments?

It should be acknowledged that eye dominance, like any lateralization, is not a dichotomic phenomenon. Rather, it has been repeatedly suggested that eye dominance is a continuous measure^83–85^. Notably, Carey and Hutchinson^86^ suggested that the strength of eye dominance might be revealed by the eccentricity at which the participants switch from one eye to the other in a task requiring visual alignments with different target eccentricities (see also^87^). We also previously showed that eye dominance influence on reaction times can vary according to eye dominance strength determined through saccadic peak velocity analysis^18^. Further studies should explore the influence of the eye dominance strength on saccade curvature parameters, and more generally on visual processing.

Finally, we think that results supporting the presence of a visual bias related to the eye dominance (present results and previous ones^16–18,22,23^ should be taken into account when considering several previously described phenomena. For example, Ossandon et al.^88^ reported a clear leftward bias in image visual exploration. Given the proportion of right eye dominance participants in a random population (66% according to^6^; see also^89^) and the contralateral visual bias observed here, Ossandon et al.’s results could, at least in part, be explained by the eye dominance of participants. Saccade trajectory is also often studied as a proxy to evaluate different cognitive processes (e.g.^71,90,91^). Here also, taking eye dominance into account could explain part of the variability in the results and help draw clearer conclusions.

To conclude, the present study was designed to further test the hypothesis of a contralateral visual bias with respect to DE. We indeed observed a larger curvature of the trajectory of vertical saccades toward a distractor when it was presented in the contralateral hemifield with respect to the DE, both in binocular and monocular conditions. These results confirm that the eye dominance represents a lateralization of the visual system that introduces a contralateral bias with sensorimotor consequences. More research is needed to explore the consequences of this lateralization on higher cognitive skills.

## Supplementary Information

Supplementary Figure 1.

Supplementary Figure 2.

Supplementary Figure 3.

## Data Availability

The data that support the findings of the study are available from the corresponding author upon reasonable request.
