# Supplementary material for: Distractor-induced saccade trajectory curvature reveals visual contralateral bias with respect to the dominant eye
Source: Sci Rep. 2022 Dec 16;12:21737. doi: 10.1038/s41598-022-26319-3 (PMC9758137; doi:10.1038/s41598-022-26319-3)
Supplement: Supplementary file 1 — Supplementary Figure 1. [file 41598_2022_26319_MOESM1_ESM.pdf]

Binocular / Two Distractors

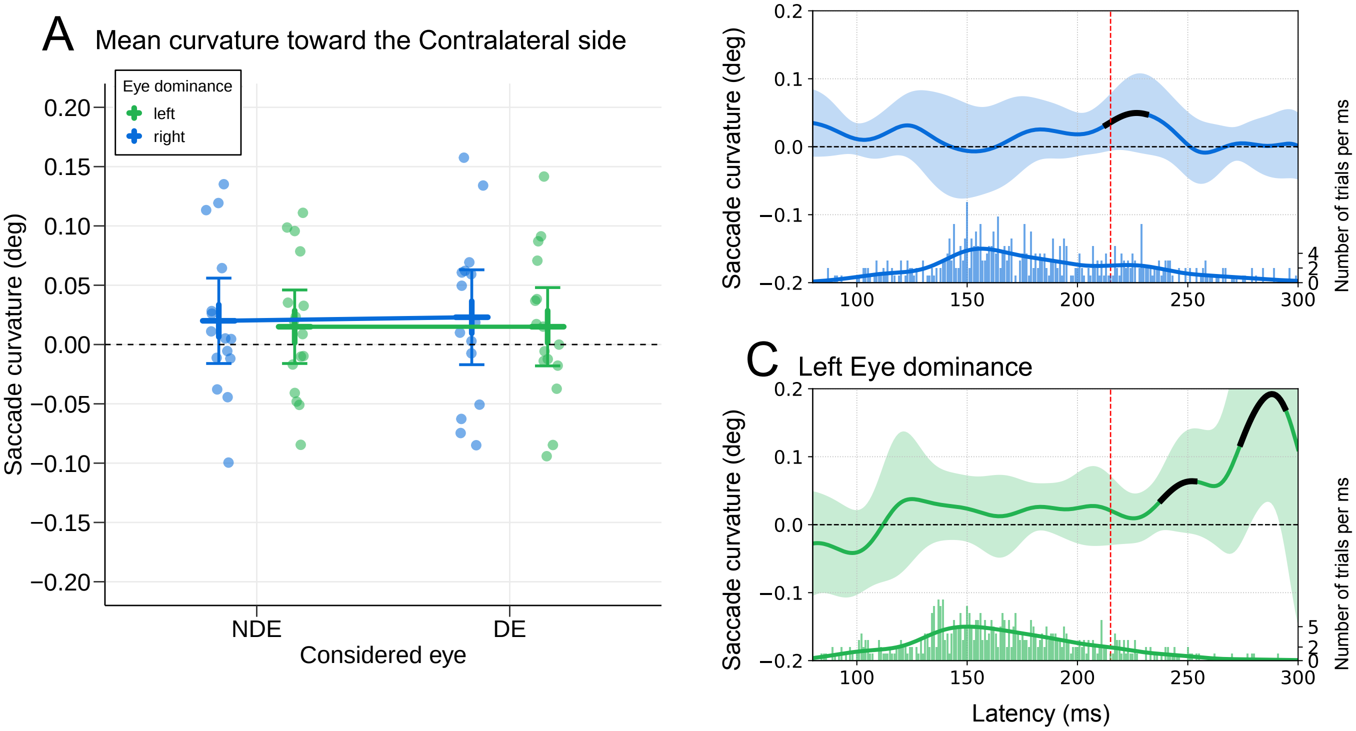

Supplementary Material - Figure 1. Saccade trajectory curvature in Binocular / Two Distractors condition. A. For each participant and for saccades initiated before the toward/away switch latency (215ms, red vertical line on Figure 3), the saccade curvature values when two distractors were presented was plotted as a function of the considered eye. Positive values correspond to a curvature toward the distractor placed in the contralateral hemifield. Error bars represent 95% confidence intervals. The linear model approach shows a satistically significant difference from zero ($p < 0.05$) for the whole population. See text for further details. **B.** Saccade curvature as a function of saccade latency in right DE participants. Positive values correspond to a curvature toward the distractor placed in the contralateral hemifield. Data are smoothed with a Gaussian kernel. Black line segment corresponds to a cluster with significant weighted within-subjects t-tests with respect to zero ($p < 0.05$) for successive time points, but this cluster did not survive at the multiple comparisons control and thus should be considered as non-significant. The shaded areas are 95% within-subjects confidence intervals. The histogram at the bottom of the graph shows the number of trials per 1 millisecond bin (right axis, line corresponds to smoothing with the same kernel as above). **C.** Saccade curvature as a function of saccade latency in left DE participants. Same organization as for pannel B. Black line segments correspond to clusters with significant t-tests from zero for successive time points, but none of these clusters survive at the multiple comparisons control and thus should be considered as non-significant.
