# Supplementary material for: Distractor-induced saccade trajectory curvature reveals visual contralateral bias with respect to the dominant eye
Source: Sci Rep. 2022 Dec 16;12:21737. doi: 10.1038/s41598-022-26319-3 (PMC9758137; doi:10.1038/s41598-022-26319-3)
Supplement: Supplementary file 2 — Supplementary Figure 2. [file 41598_2022_26319_MOESM2_ESM.pdf]

Monocular / One Distractor

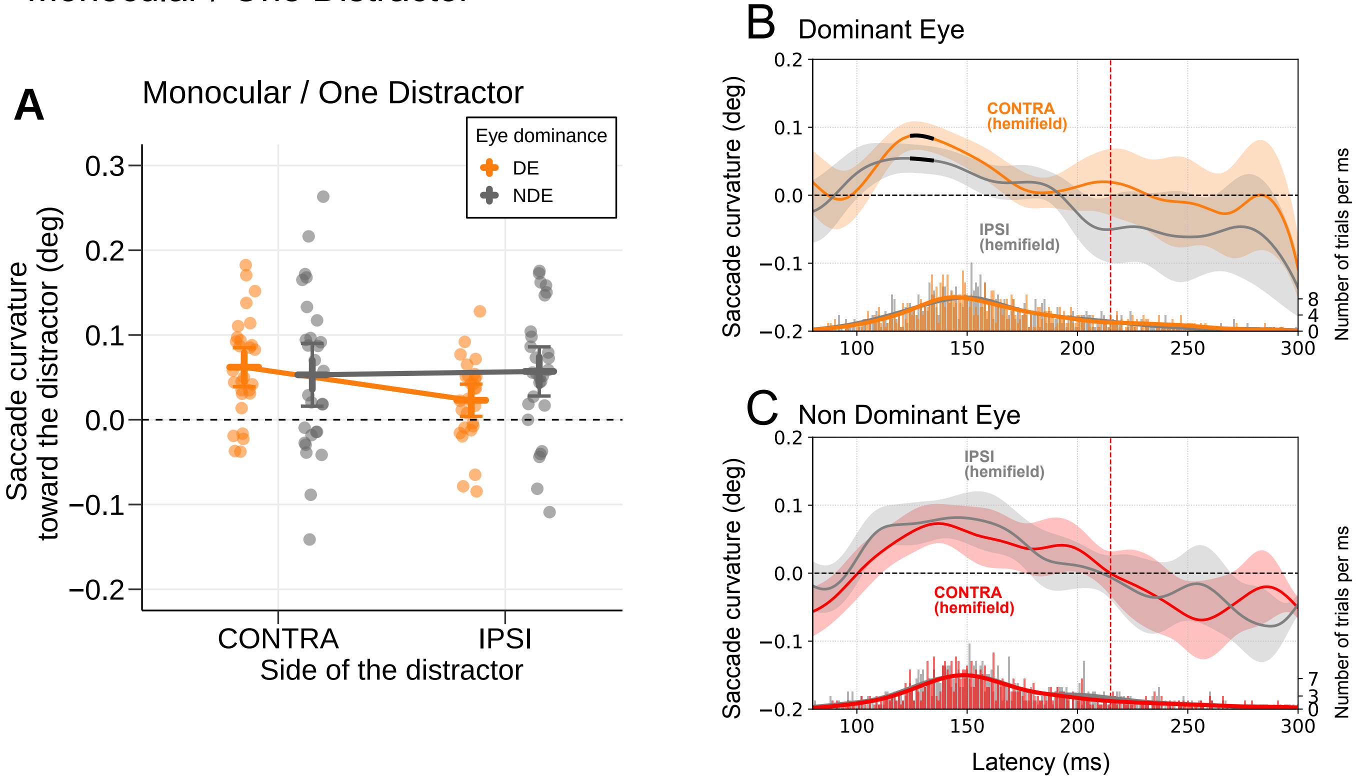

Supplementary Material - Figure 2. Saccade trajectory curvature in Monocular / One Distractor. A. For both Left and Right eye dominance groups, saccade curvature was measured for contralateral and ipsilateral distractors with respect to the Dominant Eye. **B.** Saccade curvature as a function of saccade latency for the Dominant Eye. Orange and grey curves correspond to curvature values for trials with a distractor in the contralateral or ipsilateral hemifield, respectively. Positive values correspond to a curvature toward the distractor. Data are smoothed with a Gaussian kernel. The shaded areas are 95% within-subjects confidence intervals. The bottom histogram displays the number of trials per 1 millisecond bin for both conditions (right axis, lines correspond to smoothing with the same kernel as above). Black line segment corresponds to a cluster with significant t-tests between the two hemifields for successive time points, but this cluster does not survive at the multiple comparisons control and thus should be considered as non-significant. **C.** Saccade curvature as a function of saccade latency for the Non Dominant Eye. Red (arbitrary color) and grey curves correspond to curvature values for trials with a distractor in the contralateral or ipsilateral hemifield, respectively. Same organization as for pannel B.
