# Supplementary material for: Distractor-induced saccade trajectory curvature reveals visual contralateral bias with respect to the dominant eye
Source: Sci Rep. 2022 Dec 16;12:21737. doi: 10.1038/s41598-022-26319-3 (PMC9758137; doi:10.1038/s41598-022-26319-3)
Supplement: Supplementary file 3 — Supplementary Figure 3. [file 41598_2022_26319_MOESM3_ESM.pdf]

Monocular / Two Distractors

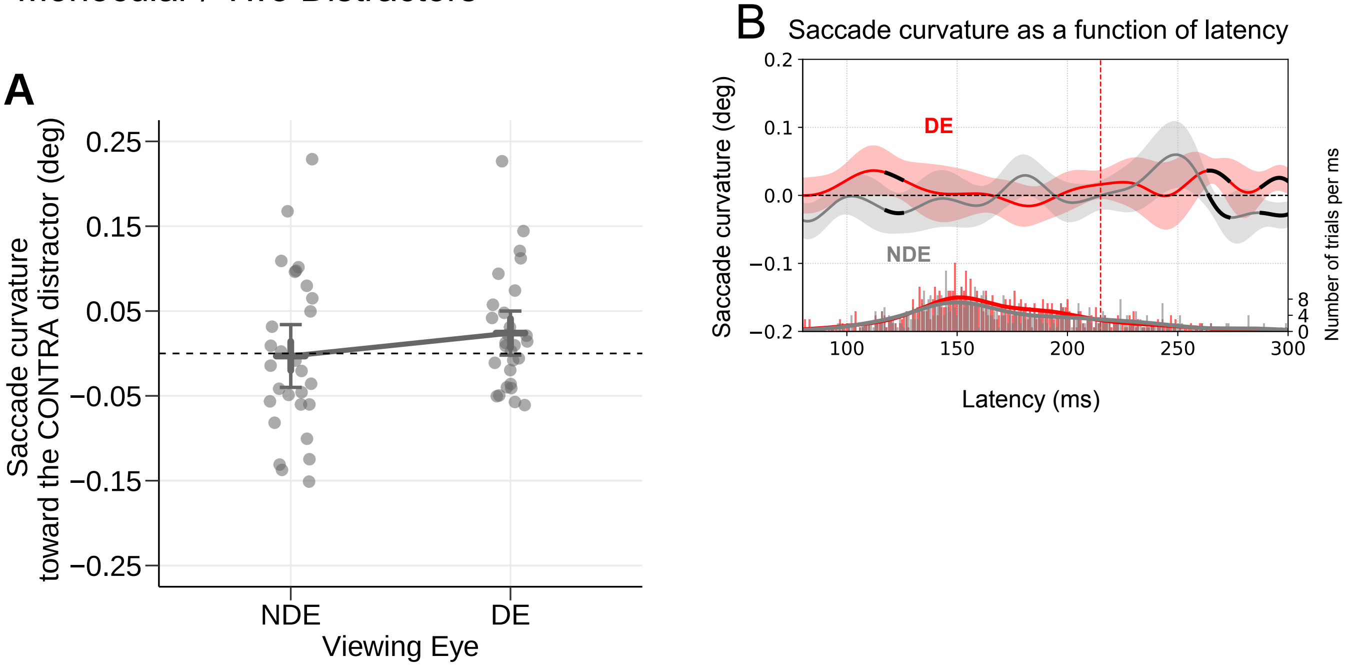

Supplementary Material - Figure 3. Saccade trajectory curvature in Monocular / Two Distractors. A. For both Left and Right eye dominance groups, saccade deviation was considered as positive when toward the distractor in the contralateral hemifield with respect to the DE. Error bars represent 95% confidence intervals. **B.** Red (arbitrary color) and grey curves correspond to curvature values for the Dominant Eye and the Non Dominant Eye, respectively. Positive values correspond to a curvature toward the distractor placed in the contralateral hemifield. Data are smoothed with a Gaussian kernel. The shaded areas are 95% within-subjects confidence intervals. The bottom histogram displays the number of trials per 1 millisecond bin for both conditions (right axis, lines correspond to smoothing with the same kernel as above). Black line segments correspond to clusters with significant t-tests between the two conditions for successive time points, but none of these clusters survive at the multiple comparisons control ans thus should be considered as non-significant.
